# Brain-like optical computing: a pathway from edge intelligence to embodied intelligence

**DOI:** 10.1093/nsr/nwag336

**Published:** 2026-06-04

**Authors:** Yuetian Jia, Xinyuan Fang, Min Gu

**Affiliations:** School of Artificial Intelligence Science and Technology, University of Shanghai for Science and Technology, China; Institute of Photonic Chips, University of Shanghai for Science and Technology, China; School of Artificial Intelligence Science and Technology, University of Shanghai for Science and Technology, China; Institute of Photonic Chips, University of Shanghai for Science and Technology, China; School of Artificial Intelligence Science and Technology, University of Shanghai for Science and Technology, China; Institute of Photonic Chips, University of Shanghai for Science and Technology, China

## Abstract

Moving beyond matrix-based optics, brain-like optical computing combines photonic memory, learning and event-driven processing for ultralow-latency perception, high-bandwidth interaction and energy-efficient autonomous control across edge and embodied systems.

The rapid expansion of artificial intelligence is reshaping the frontier of computation from centralized model training toward intelligent systems that must operate directly in the physical world. This transition is most evident in edge intelligence and embodied intelligence, where machines are required not only to infer, but also to perceive, decide, and act in real time under strict constraints of latency, power, and local autonomy. In such scenarios, response times often need to lie within the microsecond to millisecond regime; power budgets are frequently limited to milliwatt levels; and high-bandwidth sensor data must be processed locally to avoid communication overhead, privacy risks, and network dependence. However, these demands are increasingly difficult to satisfy with conventional electronic architectures, where memory access overhead, data movement, and energy dissipation dominate system performance. These limitations motivate brain-like optical computing (BOC), an emerging paradigm that seeks to realize intelligence through the physical dynamics of light itself. By harnessing propagation, interference, nonlinearity, and optical memory within a unified substrate, BOC enables sensing, processing, and decision making to be more tightly integrated than in architectures composed of separated functional modules. Owing to the intrinsic properties of light, including ultrafast propagation, massive parallelism, and high-capacity multiplexing, representative optical implementations suggest the potential for picosecond–nanosecond response, Gb/s–Tb/s bandwidth and femtojoule-level operation [1–3]. The embedded table in Fig. 1 quantitatively compares brain-like electronic computing (BEC) [4] and BOC [5] in terms of system response latency, transmission/computation bandwidth, and energy per multiply-accumulate operation. Rather than serving merely as another accelerator for existing AI workloads, BOC offers a physically matched pathway for future edge and embodied intelligence systems, where

**Figure 1.**
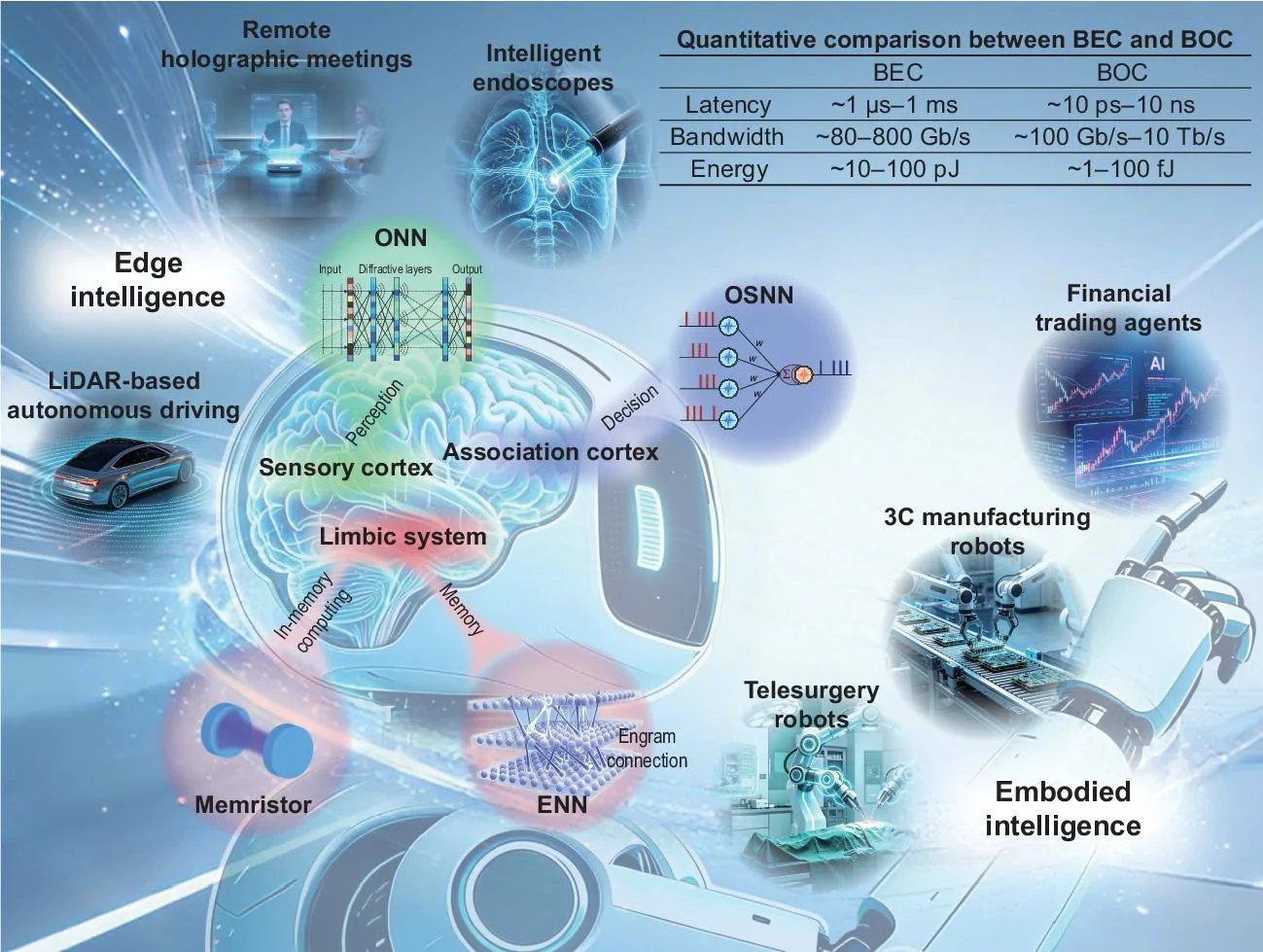
Conceptual illustration of BOC, showing a unified photonic architecture from edge intelligence to embodied intelligence. ONNs serve as multimodal perception modules analogous to the sensory cortex, OSNNs function as information-processing and decision modules analogous to the association cortex, while ENNs and optical memristors provide memory, state reconfiguration, and synaptic plasticity analogous to the limbic system (for example, hippocampus and amygdala). Together, these components form an integrated brain-like optical substrate supporting low-latency, high-bandwidth, and energy-efficient intelligent operation across representative future applications.

**Table 1.** Comparative overview of key technologies for BOC.

Technology	Features	Advantages	Challenges
ONNs	Optical matrix processing	High speed, energy efficient	Weak nonlinearity, limited dynamics
OSNNs	Temporal/event-driven processing	Temporal coding, sparse operation	System complexity, noise accumulation, training scalability
ENNs	State-based learning and memory	Adaptive memory, rich states	Early exploration stage, no standards training
Optical memristors	In-memory computing	Nonvolatile, plastic, intrinsic nonlinearity	Stability, large-scale integration

ultralow latency, high bandwidth perception, and energy efficient control are simultaneously required.

BOC does not emerge in isolation, but represents the latest stage in the long evolution of optical computing. Early efforts in the 1960s and 1970s focused on analog Fourier optics for signal processing and pattern recognition, followed in the 1980s and 1990s by optical correlators and hybrid optoelectronic processors that exploited parallelism for specialized tasks [1]. More recently, the resurgence of AI has renewed interest in photonic hardware through optical neural networks (ONNs), which use diffraction, interference, and modal transformations to accelerate matrix-vector operations [2]. These advances established optics as a powerful computational substrate and demonstrated compelling advantages in speed and energy efficiency. However, most existing architectures remain centered on linear feedforward transforms and static inference pipelines. As a result, they are better viewed as optical accelerators than complete intelligent systems. Existing platforms, including Mach–Zehnder interferometer meshes and diffractive deep neural networks (D^2^NNs), therefore mark an important milestone, but do not yet capture the adaptive, stateful, and closed-loop behaviors required for physical intelligence [1,2].

The next step is to move beyond computation-centric photonics toward native intelligence-centric photonics that aims to achieve capabilities similar to biological intelligence, embodying temporal dynamics, event-driven responses, continual memory formation, and adaptive interaction with changing environments. These requirements call for optical substrates where perception, neural dynamics, learning, and memory are physically embodied rather than externally emulated. One prominent direction is optical spiking neural networks (OSNNs), where information is encoded in the precise timing of optical pulses. By leveraging ultrafast modulators, microring resonators, and nonlinear materials, OSNNs can implement thresholding, temporal integration, and pulse generation directly in the optical domain, enabling event-driven processing and rich temporal computation [3,6]. Compared with ONNs, OSNNs provide richer temporal dynamics, but still face challenges in system complexity, noise, and scalable training. Complementing temporal intelligence, engram neural networks (ENNs) represent a cognition-inspired memory framework in which learning occurs through the formation and reactivation of distributed memory engrams, allowing experience to be stored as persistent network states rather than solely in static parameters [7]. On photonic platforms, such behavior may be supported by phase-change materials, optical memristors, and reconfigurable interferometric or resonant networks that enable persistent memory encoding and history-dependent plasticity. At the device level, optical memristors further provide nonvolatile storage, synaptic plasticity, and in-memory computing through reversible optical-state modulation [8], while ongoing challenges in stability and large-scale integration. Together with ONN-based perception front ends, these technologies define a functional architecture in which sensing, memory, reasoning, and action can coexist within a unified photonic substrate. A comparative overview of these technological routes is summarized in Table 1.

Beyond neuromorphic photonics [9,10] which primarily emphasizes brain-inspired computing modules for efficient information processing, BOC adopts a broader system-level perspective aimed at deployable optical intelligent platforms with integrated capabilities of perception, memory, reasoning, and autonomous action. This translational orientation makes BOC particularly relevant to both edge and embodied intelligence, where sensing, computation, and response must occur locally under stringent constraints of latency, bandwidth, and energy consumption. For edge intelligence, BOC can reduce reliance on cloud infrastructure and improve responsiveness, privacy, and system robustness in applications such as (i) LiDAR-based autonomous driving where rapid obstacle detection and trajectory planning are required, (ii) remote holographic meetings where high-dimensional visual data must be processed and transmitted with minimal delay, and (iii) intelligent endoscopes where compact medical devices require energy-efficient *in-situ* image analysis under strict power and thermal constraints [11]. For embodied intelligence, BOC is promising for (i) telesurgery robots where ultralow latency is essential for precise manipulation and haptic feedback, (ii) 3C manufacturing robots where high-bandwidth parallel sensing supports visual inspection, motion coordination, and adaptive assembly, and (iii) financial trading agents where continuous market perception and rapid decision-making are critical (Fig. 1). Among potential physical realizations, D^2^NN architectures offer a compatible implementation by embedding computation into optical wave propagation and trainable wavefront modulation, enabling massively parallel processing without iterative memory access. Recent optical generative models further demonstrate that complex generative tasks can emerge directly within D^2^NN architectures, extending their role from deterministic inference to *in situ* physical-layer information processing [12]. In parallel, integration with optical spiking processors, meta-imaging systems or vertical-cavity surface-emitting laser arrays [13] point toward compact, high-efficiency, and low-power intelligent modules. As these photonic processors become increasingly integrated with sensors, structural materials, joints, and actuation units, BOC may enable unified perception–decision–action loops executed directly within the optical domain, providing a promising physical framework for next-generation intelligent systems.

Despite its promise, BOC faces challenges across multiple layers of development with different priorities and time scales. At the first level (brain-optics integration), the key challenge is not simply reproducing existing neural networks, but identifying native optical mechanisms for perception, memory, adaptation, and decision-making. A critical direction is to develop physics-native intelligence models, in which computation emerges directly from optical dynamics such as interference, temporal oscillation, nonlinear attractors, and distributed memory states. This includes dynamical system-based approaches such as photonic reservoir computing, delay-based feedback systems, oscillator, and synchronization networks which provide multiple physically grounded routes toward stateful and adaptive computation. At the second level (hardware implementation), large-scale BOC systems require robust phase, wavelength, and modal control while maintaining plasticity and low loss. This spans multiple device and integration technologies, including reconfigurable metasurfaces and programmable diffractive optics for wavefront control, phase-change materials, and optical memristive devices for nonvolatile memory and plasticity, integrated optical spiking elements based on microrings, excitable lasers or electro-optic modulation, as well as heterogeneous integration of silicon photonics with active materials for scalable functionality. At the third level (application deployment), practical BOC systems must support real-time interaction with dynamic environments. Rather than static benchmarking alone, future platforms should adopt closed-loop task evaluation in edge intelligence and embodied systems. This involves system-level co-design of sensing, computation, and actuation, including in-sensor computing architectures, integrated optical perception pipelines such as LiDAR-based processing, and real-time perception-control loops for robotics, together with enabling technologies such as compact photonic packaging, standardized evaluation metrics, and scalable photonic-electronic co-integration to bridge laboratory prototypes and real-world deployment.

Unlocking the full potential of BOC will require coordinated advances across disciplines as well as a coherent strategic framework. At the technology level, progress in information science, brain science, and materials science must converge to establish a unified physical basis for optical intelligence. Within this broader context, quantum photonic computing [14] based on superposition, entanglement, and unitary evolution may extend information encoding capacity, enable distributed processing via non-classical correlations, and support remote coordination among spatially separated edge nodes. These capabilities could facilitate low-latency collaborative control, secure information sharing, and more efficient large-scale deployment for future edge intelligence systems. At the system level, hybrid architectures ranging from optical-electronic [15] to all-optical systems [16], are expected to provide a versatile platform for integrating complementary computational modalities, enabling efficient, high-speed, and reconfigurable deployment of BOC. At the ecosystem level, long-term coordination among research institutions, industry, and government will be essential for progress in fabrication infrastructure, packaging technologies, standardized testing, and interdisciplinary training. As these foundations mature, BOC will progress from prototypes to deployable technologies, harnessing the physics of light to enable autonomous, adaptive, and continuously learning intelligent systems that define the next era of physical intelligence.
